# Supplementary material for: Functional metagenomics-guided discovery of potent Cas9 inhibitors in the human microbiome
Source: eLife. 2019 Sep 10;8:e46540. doi: 10.7554/eLife.46540 (PMC6739867; doi:10.7554/eLife.46540)
Supplement: Supplementary file 1. [file elife-46540-supp1.docx]

**Table S1**. False positive genotypes and frequencies encountered during the development of the SpyCas9 Acr selection. Asterisks (*) indicate genotyped experiments that are referenced in Figure 1—figure supplement 2. Parentheses indicate the number of colonies queried and the number with the indicated genotype.

| **Experiment** | **Target Plasmid** | **pSpyCas9 used**  (table S6) | **Cas9 expression** | **No. of target sites** | **Iteration No.** | **pSpyCas9 genotype** | **Target site genotype** | **Surviving Proportion of Kan^R^ Transformants** | **Notes** |
| --- | --- | --- | --- | --- | --- | --- | --- | --- | --- |
| A | pZE21-GFP | pSpyCas9_crA | Constitutive | 1 | 1 | loss-of-function mutant (8/8) | wild-type (8/8) | not determined | Constitutive SpyCas9 restricted transformation |
| B | pZE21-GFP | pSpyCas9_crZ | Constitutive | 1 | 1 | loss-of-function mutant (6/6) | wild-type (6/6) | not determined | Constitutive SpyCas9 restricted transformation |
| C* | pZE21-GFP | pSpyCas9_in_crA | Inducible | 1 | 1 | loss-of-function mutant (6/6) | wild-type (6/6) | 5.05E-06 | In Figure 1—figure supplement 2 |
| D | pZE21-GFP | pSpyCas9_in_crA | Inducible | 1 | 1 | no colonies tested | no colonies tested | 1.87E-05 | In Figure 1—figure supplement 2 |
| E* | pZE21-GFP | pSpyCas9_in_crA | Inducible | 1 | 2 | wild-type (6/6) | PAM or protospacer mutant (12/12) | 0.03 | In Figure 1—figure supplement 2 |
| F | pZE21-GFP | pSpyCas9_in_crA | Inducible | 1 | 1 | no colonies tested | no colonies tested | 4.82E-05 | In Figure 1—figure supplement 2 |
| G* | pZE21-GFP | pSpyCas9_in_crAcrZ | Inducible | 2 | 1 | loss-of-function mutant (11/11) | wild-type (10/10) | 7.03E-06 | In Figure 1—figure supplement 2 |
| H | pZE21-GFP | pSpyCas9_in_crA | Inducible | 1 | 1 | no colonies tested | no colonies tested | 2.98E-05 | In Figure 1—figure supplement 2 |
| I | pZE21-GFP | pSpyCas9_in_crAcrZ | Inducible | 2 | 1 | no colonies tested | no colonies tested | 1.06E-05 | In Figure 1—figure supplement 2 |
| J* | pZE21-GFP | pSpyCas9_in_crA | Inducible | 1 | 2 | loss-of-function mutant (4/4) | wild-type (8/8) | 2.72E-05 | In Figure 1—figure supplement 2 |
| K* | pZE21-GFP | pSpyCas9_in_crAcrZ | Inducible | 2 | 2 | loss-of-function mutant (6/6) | wild-type (9/9) | 3.44E-06 | In Figure 1—figure supplement 2 |
| L* | pZE21-GFP | pSpyCas9_in_crAcrZ | Inducible | 2 | 2 | loss-of-function mutant (6/6) | wild-type (20/20) | 1.46E-05 | In Figure 1—figure supplement 2 |
| M | pZE21-GFP | pSpyCas9_in_crAcrZ | Inducible | 2 | 1 | no colonies tested | no colonies tested | 4.20E-05 | In Figure 1—figure supplement 2 |
| N | pZE21-GFP | pSpyCas9_in_crAcrZ | Inducible | 2 | 2 | no colonies tested | no colonies tested | 3.77E-04 | In Figure 1—figure supplement 2 |
| O | pZE21-GFP | pSpyCas9_in_crAcrB | Inducible | 2 | 1 | no colonies tested | no colonies tested | 3.75E-05 | In Figure 1—figure supplement 2 |
| P | pZE21-GFP | pSpyCas9_in_crAcrB | Inducible | 2 | 2 | no colonies tested | no colonies tested | 1.27E-04 | In Figure 1—figure supplement 2 |

**Table S2**. Metagenomic library information. Each library has an average DNA insert size of ~2 Kb (Clemente et al., 2015; Pehrsson et al., 2016). In calculating the number of clones subjected to SpyCas9 selection, we assumed that transformation is a Poisson process.

| **Library Information** | | **Iteration #1** | | | **Iteration #2** | | |
| --- | --- | --- | --- | --- | --- | --- | --- |
| **Library** | **Unique Clones (x10^6^)** | **Transformants (x10^6^)** | **Unique Clones Subjected to SpyCas9 Selection (x10^6^)** | **KanR Colonies Collected** | **Amt. Transformed (ng)** | **Transformants (x10^6^)** | **Titer Volumes Collected** |
| Oral_3 | 3.65 | 5 | 2.72 | 2850 | 272 | 56 | 2ml / 100µl |
| Oral_5 | 3.88 | 7.85 | 3.37 | 3580 | 396 | 71.8 | 100µl / 1µl |
| Fecal_01A | 1.65 | 21.4 | 1.65 | 3110 | 261 | 32.9 | 1µl / 0.01µl |
| Fecal_01E | 1.35 | 4.88 | 1.31 | 520 | 236 | 29.8 | 100µl / 1µl |
| Fecal_03G | 2.7 | 4.36 | 2.16 | 2910 | 216 | 46.2 | 1µl / 0.01µl |
| GFP | n/a | 42.8 | n/a | 5650 | 308 | 3.86 | 100µl |

**Table S3**. Summary of twice-iterated functional selections for SpyCas9 Acrs. Wild-type target site statistics were determined using bulk plasmids surviving two rounds of SpyCas9 selection. The ‘ts’ acronym stands for ‘target site’, which encompasses both the protospacer and PAM sequences required for SpyCas9 restriction. RPBM stands for ‘Reads per base-pair per million reads’.

| **Sample** | **GFP +ara** | **Oral_3** | **Oral_5** | **Fecal_01A** | **Fecal_01E** | **Fecal_03G** |
| --- | --- | --- | --- | --- | --- | --- |
| % wild-type target sites (tsA/tsB) | 99.4/99.0 | 98.5/86.2 | 98.8/82.2 | 95.9/95.9 | 99.6/54.9 | 32.2/0.4 |
| Contigs assembled (titer plate 1 / 2) | n/a | 6/10 | 69/45 | 14/8 | 28/15 | 7/4 |
| Contigs with RPBM > 2 (titer plate 1 / 2) | n/a | 3/6 | 21/19 | 7/6 | 16/8 | 2/1 |
| Final unique contig count | n/a | 3 | 20 | 10 | 17 | 1 |

**Table S4**. All contigs in the final dataset. Contigs are organized by library. Reads per base-pair per million reads (RPBM) indicate coverage for a given contig, which is a proxy for that sequence’s abundance following SpyCas9 selection. Proteins with close homologs in a predicted phage were used to classify a given contig as phage-associated (see methods). NCBI taxa were determined by blastn and blastp searches of contig and protein sequences; GTDB taxa were determined as described in the methods and were used throughout most of the manuscript to resolve a well-recognized polyphyly among the Clostridiales.

| **Library** | **Contig** | **GenBank Acc. No** | **RPBM** | **Phage-Associated?** | **NCBI Order** | **NCBI Genus** | **GTDB Genus** | **Cas9 in GTDB Genus?** |
| --- | --- | --- | --- | --- | --- | --- | --- | --- |
| Oral_3 | O3_1 | MK637558 | 5.45 | No | Enterobacteriales | Escherichia | Escherichia | No |
| Oral_3 | O3_2 | MK637556 | 4.31 | No | Bacteroidales | Porphyromonas | Porphyromonas | **Yes** |
| Oral_3 | O3_3 | MK637557 | 9.60 | No | Bacteroidales | Porphyromonas | Porphyromonas | **Yes** |
| Oral_5 | O5_1 | MK637560 | 8.15 | **Yes** | Actinomycetales | Actinomyces | Actinomyces | **Yes** |
| Oral_5 | O5_2 | MK637561 | 7.21 | **Yes** | Enterobacteriales | Escherichia | Escherichia | No |
| Oral_5 | O5_3 | MK637562 | 29.20 | No | Neisseriales | Neisseria | Neisseria | **Yes** |
| Oral_5 | O5_4 | MK637563 | 13.74 | No | Bacteroidales | Porphyromonas | Porphyromonas | **Yes** |
| Oral_5 | O5_5 | MK637564 | 4.31 | No | Neisseriales | Neisseria | Neisseria | **Yes** |
| Oral_5 | O5_6 | MK637565 | 184.49 | **Yes** | Bacteroidales | Porphyromonas | Porphyromonas | **Yes** |
| Oral_5 | O5_7 | MK637566 | 371.30 | **Yes** | Neisseriales | Neisseria | Neisseria | **Yes** |
| Oral_5 | O5_8 | MK637567 | 85.44 | No | Neisseriales | Neisseria | Neisseria | **Yes** |
| Oral_5 | O5_9 | MK637568 | 69.77 | No | Bacteroidales | Prevotella | Prevotella | **Yes** |
| Oral_5 | O5_10 | MK637571 | 4.15 | No | Bacteroidales | Prevotella | Prevotella | **Yes** |
| Oral_5 | O5_11 | MK637572 | 7.09 | **Yes** | Burkholderiales | Unknown | Unknown | n/a |
| Oral_5 | O5_12 | MK637569 | 4.25 | No | Neisseriales | Neisseria | Neisseria | **Yes** |
| Oral_5 | O5_13 | MK637570 | 2.94 | No | Selenomonadales | Selenomonas | Centipeda | No |
| Oral_5 | O5_14 | MK637575 | 9.97 | No | Neisseriales | Neisseria | Neisseria | **Yes** |
| Oral_5 | O5_15 | MK637576 | 2.90 | No | Bacteroidales | Alloprevotella | Alloprevotella | **Yes** |
| Oral_5 | O5_16 | MK637573 | 8.32 | No | Lactobacillales | Streptococcus | Streptococcus | **Yes** |
| Oral_5 | O5_17 | MK637574 | 4.93 | No | Neisseriales | Kingella | Kingella | **Yes** |
| Oral_5 | O5_18 | MK637577 | 2.50 | No | Neisseriales | Neisseria | Neisseria | **Yes** |
| Oral_5 | O5_19 | MK637578 | 93.26 | No | Bacteroidales | Alloprevotella | Alloprevotella | **Yes** |
| Oral_5 | O5_20 | MK637559 | 29.47 | No | Bacteroidales | Alloprevotella | Alloprevotella | **Yes** |
| Fecal_01A | F01A_1 | MK637584 | 3.28 | **Yes** | Clostridiales | Ruminococcus | Ruminococcus_E | **Yes** |
| Fecal_01A | F01A_2 | MK637582 | 766.28 | **Yes** | Clostridiales | Clostridium | Clostridium_Q | **Yes** |
| Fecal_01A | F01A_3 | MK637583 | 2.54 | **Yes** | Clostridiales | Coprococcus | Coprococcus | **Yes** |
| Fecal_01A | F01A_4 | MK637587 | 83.66 | **Yes** | Clostridiales | Clostridium | CAG-217 | No |
| Fecal_01A | F01A_5 | MK637588 | 3.67 | No | Bacteroidales | Bacteroides | Bacteroides | **Yes** |
| Fecal_01A | F01A_6 | MK637585 | 10.91 | No | Clostridiales | Roseburia | Roseburia | **Yes** |
| Fecal_01A | F01A_7 | MK637586 | 2.25 | No | Coriobacteriales | Collinsella | Collinsella | **Yes** |
| Fecal_01A | F01A_8 | MK637580 | 3.43 | No | Clostridiales | Ruminococcus | Ruminococcus_B | **Yes** |
| Fecal_01A | F01A_9 | MK637581 | 4.95 | No | Clostridiales | Unknown | KLE1615 | No |
| Fecal_01A | F01A_10 | MK637579 | 3.26 | No | Bacteroidales | Bacteroides | Bacteroides | **Yes** |
| Fecal_01E | F01E_1 | MK637593 | 6.72 | No | Bifidobacteriales | Bifidobacterium | Bifidobacterium | **Yes** |
| Fecal_01E | F01E_2 | MK637589 | 20.76 | No | Bifidobacteriales | Bifidobacterium | Bifidobacterium | **Yes** |
| Fecal_01E | F01E_3 | MK637590 | 6.36 | No | Bifidobacteriales | Bifidobacterium | Bifidobacterium | **Yes** |
| Fecal_01E | F01E_4 | MK637600 | 5.85 | No | Bacteroidales | Bacteroides | Bacteroides | **Yes** |
| Fecal_01E | F01E_5 | MK637601 | 2.89 | No | Bifidobacteriales | Bifidobacterium | Bifidobacterium | **Yes** |
| Fecal_01E | F01E_6 | MK637598 | 3.05 | No | Bifidobacteriales | Bifidobacterium | Bifidobacterium | **Yes** |
| Fecal_01E | F01E_7 | MK637599 | 2.73 | No | Enterobacteriales | Escherichia | Escherichia | No |
| Fecal_01E | F01E_8 | MK637597 | 239.49 | No | Bifidobacteriales | Bifidobacterium | Bifidobacterium | **Yes** |
| Fecal_01E | F01E_9 | MK637591 | 3.04 | No | Enterobacteriales | Escherichia | Escherichia | No |
| Fecal_01E | F01E_10 | MK637596 | 46.35 | No | Enterobacteriales | Escherichia | Escherichia | No |
| Fecal_01E | F01E_11 | MK637592 | 6.75 | No | Bacteroidales | Bacteroides | Bacteroides | **Yes** |
| Fecal_01E | F01E_12 | MK637605 | 463.50 | No | Bifidobacteriales | Bifidobacterium | Bifidobacterium | **Yes** |
| Fecal_01E | F01E_13 | MK637595 | 2.01 | No | Bifidobacteriales | Bifidobacterium | Bifidobacterium | **Yes** |
| Fecal_01E | F01E_14 | MK637594 | 2.96 | No | Bifidobacteriales | Bifidobacterium | Bifidobacterium | **Yes** |
| Fecal_01E | F01E_15 | MK637603 | 151.77 | No | Bifidobacteriales | Bifidobacterium | Bifidobacterium | **Yes** |
| Fecal_01E | F01E_16 | MK637602 | 51.89 | **Yes** | Bifidobacteriales | Bifidobacterium | Bifidobacterium | **Yes** |
| Fecal_01E | F01E_17 | MK637604 | 15.19 | No | Bacteroidales | Bacteroides | Bacteroides | **Yes** |
| Fecal_03G | F03G_1 | MK637606 | 2.06 | No | Bifidobacteriales | Bifidobacterium | Bifidobacterium | **Yes** |

**Table S5**. Genera with homologs of type II-A Acrs, as depicted in Figure 4—figure supplement 2.

| **Type II Acr** | **Query Sequence** | **GTDB Genera** |
| --- | --- | --- |
| AcrIIA1 | MTIKLLDEFLKKHDLTRYQLSKLTGISQNTLKDQNEKPLNKYTVSILRSLSLISGLSVSDVLFELEDIEKNSDDLAGFKHLLDKYKLSFPAQEFELYCLIKEFESANIEVLPFTFNRFENEEHVNIKKDVCKALENAITVLKEKKNELL | Listeria, Lactobacillus_D |
| AcrIIA2 | MTLTRAQKKYAEAMHEFINMVDDFEESTPDFAKEVLHDSDYVVITKNEKYAVALCSLSTDECEYDTNLYLDEKLVDYSTVDVNGVTYYINIVETNDIDDLEIATDEDEMKSGNQEIILKSELK | Listeria |
| AcrIIA3 | MFNKAEIMKQAWNWFNDSNIWLSDIEWVSYTDKEKSFSVCLKAAWSKAKEEVEESKKESKHIAKSEELKAWNWAERKLGLHFNISDDEKFTSVKDETKINFGLSVWACAMKAVKLHNDLFPQTAA | Listeria, Streptococcus, Enterococcus_A |
| AcrIIA4 | MNINDLIREIKNKDYTVKLSGTDSNSITQLIIRVNNDGNEYVISESENESIVEKFISAFKNGWNQEYEDEEEFYNDMQTITLKSELN | Listeria |
| AcrIIA5 | MAYGKSRYNSYRKRSFNRSNKQRREYAQEMDRLEKAFENLDGWYLSSMKDSAYKDFGKYEIRLSNHSADNKYHDLENGRLIVNIKASKLNFVDIIENKLDKIIEKIDKLDLDKYRFINATNLEHDIKCYYKGFKTKKEVI | Streptococcus, Lactobacillus_B, Granulicatella, Dolosigranulum |
| AcrIIA6 | MKINDDIKELILEYMSRYFKFENDFYKLPGIKFTDANWQKFKNGGTDIEKMGAARVNAMLSCLFEDFELAMIGKAQTNYYIDNSLKLNMPFYAYYDMFKKQLLINWLKNNRDDVICGTGRMYTASGNYIANAYLEVALESSRLGGGEYMLQMRFKNYSRSQEPIPSGRQNRLEWIENNLENIR | Streptococcus |
| AcrIIA7 | MTFGQALESLKRGHLVARKGWNGKGMFIFMRPEDSLPTNMIVNQVKSLPESFKRWVANNHGDSETDRIKFTAYLCMKAADGTIVNGWLASQTDMLANDWVIVE | Lachnospira, Ruminococcus_B, Lactococcus, Clostridium_M, Bacillus_A, TF01-11, Fusicatenibacter, Megasphaera, Anaerostipes, Lysinibacillus, Trichococcus, Paenibacillus_E, Brevibacillus_B, Caecibacter, Dehalobacter, Papillibacter, Bacillus, Bacillus_AN, Lactobacillus_F, Blautia_A, Lactobacillus_G, Lactobacillus_C, Viridibacillus, Macrococcus, Eubacterium_E, Clostridium_X, Paenibacillus, Lactobacillus_K, Bacillus_C, Paenibacillus_C, Sporomusa, Anaerosporobacter, Clostridium, Ruminococcus_D, Lactobacillus_E, Erysipelatoclostridium, Ruminococcus_C, Paenibacillus_Q, Paenibacillus_B, Tumebacillus_A, CAG-81, Clostridium_P, Eubacterium_F, Flavonifractor, Aneurinibacillus, Lactobacillus_H, Dorea, Oscillibacter, Massilibacillus, Tyzzerella, Megasphaera_A, Lactobacillus_O, UBA7182, Lactobacillus_D, Coprococcus_B, Weissella, CAG-95, Lachnotalea, Clostridium_T, Sellimonas, GCA-900066575, Enterococcus_I, Butyrivibrio, Sporosarcina_C, CAG-354, Coprococcus, Bacillus_AA, Pontibacillus, Enterococcus, Clostridium_Q, Anaerotruncus, Agathobacter, Bacillus_AD, Bacillus_AC, Cohnella, Paenibacillus_J, Eubacterium_D, Brevibacillus, UBA1033, Eubacterium_I, Desulfosporosinus, Carnobacterium, Parageobacillus |
| AcrIIA8 | MSIFTDMIPAELLINEYKKGQSGAKHDNYVSVGRIMVAIYKNNSFKNTGTVKYQDSTHSGITMSKVFIDGKEYRIDIDTQHYEVQDFDTSGRQTTLILKRIDLYG | Clostridium, Eubacterium_D, Ruminococcus_A, Ruminococcus_B |
| AcrIIA9 | MKGTEHFKQTIKEYLDGRAQTDELFAVSYAKENKNLDDCITFILNQVKASGCCGMTDDEVWSLAIHYYDEDNIDVGNPISCGVVVNHKVELTEEEKAQARKEALKAYQEEEMRKIQQRHSKPKPTAKAAQSNQTELSLFDF | Bacillus_A, Catenibacterium, UBA5454 |
| AcrIIA10 | MDNKFKLRKAINGIEELNFAFDKLTAIDYKTICRIERKMNGLSVDALADSIIASAGTRKTSSEFRIACAWVAAVKGTDGLTVDDYDQLSLDDLLELETFGLLFFVGSLE | None. |
| AcrIIA11 | MADMTLRQFCERYRKGDFLAKDRETQIEAGWYDWFCDDKALAGRLAKIWGILKGITSDYILDNYRVWFKNNCPMVGPLYDDVRFEPLDEEQRDELYFGVAIDDKRREKKYVIFTARNDYENECGFNNVREVRQFINGWEDELKNEEFYKAREKKRQEMEEANNKFAEIMQRADEILWNLKED | Acholeplasma, UBA2284, CAG-65, Ruminococcus_A, Ruminococcus_B, Dorea, Clostridium_Q, Oscillibacter, Flavinofractor, Pseudoflavinofractor, Clostridium_AJ, F23-B02 |

**Table S6**. Plasmids used in this study.

| **Plasmid** | **crRNA #1 promoter, sequence (5'-3')** | **crRNA #2 promoter, sequence (5'-3')** | **Notes** | **Refs** | **Purpose** | **Figures / Tables** |
| --- | --- | --- | --- | --- | --- | --- |
| pZE21_MCS1 | n/a | n/a | PAMs for protospacers A,Z,B,C: AGG,TGG,AGG, GAAAACT |  | Library construction, library screening, contig re-testing | Figure 1, Figure 1—figure supplement 1,2 / Tables S1, S2, S3 |
| pZE21_RBS | n/a | n/a | Deletion of multiple cloning site moves RBS near clone site | (Forsberg et al., 2015) | Express GFP in pZE21 | Figure 1, Figure 1—figure supplement 1 / Tables S1, S2, S3 |
| pZE21_tetR | n/a | n/a | Contains tetR behind pLac promoter for inducible expression of candidate Acrs |  | Acr testing | Figure 3, Figure 3—figure supplement 1, Figure 4, Figure 4—figure supplement 3, 4 |
| pSpyCas9_crA | pJ23100, GTTCATTCAGGGCACCGGAC | n/a | Addgene #48645 with crRNA_A | (Esvelt et al., 2013) | Acr selection development | Figure 1—figure supplement 1 / Table S1 |
| pSpyCas9_crZ | pJ23100, GCCCTGCAAAGTAAACTGGA | n/a | Addgene #48645 with crRNA_Z | (Esvelt et al., 2013) | Acr selection development | Figure 1—figure supplement 1 / Table S1 |
| pSpyCas9_in_crA | pJ23100, GTTCATTCAGGGCACCGGAC | n/a | pSpyCas9_crA with AraC + pBad for inducible SpyCas9 | (Lee et al., 2015) | Acr selection development, contig/ORF re-testing | Figure 1, Figure 1—figure supplement 1,2, Figure 2, Figure 3, Figure 4, / Table S1 |
| pSpyCas9_in_crA_crZ | pJ23100, GTTCATTCAGGGCACCGGAC | pJ107106, GCCCTGCAAAGTAAACTGGA | pSpyCas9_in_crA with I-SceI site and crRNA_Z |  | Acr selection development | Figure 1—figure supplement 1 / Table S1 |
| pSpyCas9_in_crA_crB | pJ23100, GTTCATTCAGGGCACCGGAC | pJ107111, GAACAAGATGGATTGCACGC | pSpyCas9_in_crA with I-SceI site and crRNA_B |  | Acr selection development, library screening | Figure 1, Figure 1—figure supplement 1,2 / Tables S1,S2,S3 |
| pSpyCas9_in_crMu | pJ23100, GTAATACTTGTCCCGCAAAG | n/a | Mu-targeting spacer for phage Mu immunity testing |  | Phage Mu immunity testing | Figure 3, Figure 3—figure supplement 1 |
| pSpyCa9_in_crNT | pJ23100, GAACGAAAAGCTGCGCCGGG | n/a | non-targeting spacer used in phage Mu immunity testing |  | Phage Mu immunity testing | Figure 3, Figure 3—figure supplement 1 |
| pTdeCas9_in_crC | pJ23100, GGTCTGACGCTCAGTGGAAC | n/a | *T. denticola cas9* and tracRNA locus from addgene #48648 cloned into pSpyCas9_in_crA. crRNA “C” then added. | (Esvelt et al., 2013) | Test AcrIIA11 breadth | Figure 4—figure supplement 4 |

**Table S7**. Oligos and gBlocks used in this study, with PCR conditions where appropriate.

| **Oligo #** | **Oligo Name** | **Sequence (5'->3')** | **Purpose** | **TM (C)** | **Ext. Time** |
| --- | --- | --- | --- | --- | --- |
| 1 | O5_3_F | CCGGGCCCCCCCTCGAGGTCAGTTATCGGCGAAGCTTCAGA | Amplify O5_3 for cloning into pZE21_MCS1 | 65 | 3.5 min |
| 2 | O5_3_R | TCAAGCTTATCGATACCGTCGTATCGATGTGCCGGAAAGC | Amplify O5_3 for cloning into pZE21_MCS1 | 65 | 3.5 min |
| 3 | O5_8_F | CCGGGCCCCCCCTCGAGGTCCTCATCCAATGGACTTTGCAC | Amplify O5_8 for cloning into pZE21_MCS1 | 61 | 3.5 min |
| 4 | O5_8_R | TCAAGCTTATCGATACCGTCTGCTCAAAAATTCAATTTACAGGG | Amplify O5_8 for cloning into pZE21_MCS1 | 61 | 3.5 min |
| 5 | O5_9_F | CCGGGCCCCCCCTCGAGGTCGGTCTAGACCTTCACTTCCATTGGATT | Amplify O5_9 for cloning into pZE21_MCS1 | 65 | 3.5 min |
| 6 | O5_9_R | TCAAGCTTATCGATACCGTCACCGTCGGCTTGCATTGAG | Amplify O5_9 for cloning into pZE21_MCS1 | 65 | 3.5 min |
| 7 | O5_14_F | CCGGGCCCCCCCTCGAGGTCGGTTCAAGCCGTTTACGGTCG | Amplify O5_14 for cloning into pZE21_MCS1 | 65 | 3.5 min |
| 8 | O5_14_R | TCAAGCTTATCGATACCGTCCGATACCGTGCGGCGTT | Amplify O5_14 for cloning into pZE21_MCS1 | 65 | 3.5 min |
| 9 | O5_16_F | CCGGGCCCCCCCTCGAGGTCCGAGGTCACCTTGGGGCAT | Amplify O5_16 for cloning into pZE21_MCS1 | 65 | 3.5 min |
| 10 | O5_16_R | TCAAGCTTATCGATACCGTCATACCGTCGTTTTTACCAGTTGATG | Amplify O5_16 for cloning into pZE21_MCS1 | 65 | 3.5 min |
| 11 | O5_19_F | CCGGGCCCCCCCTCGAGGTCCGCGTGCCTCATAGAGTTG | Amplify O5_19 for cloning into pZE21_MCS1 | 65 | 3.5 min |
| 12 | O5_19_R | TCAAGCTTATCGATACCGTCACCGTCGCAGTTGTCTTCA | Amplify O5_19 for cloning into pZE21_MCS1 | 65 | 3.5 min |
| 13 | O5_20_F | CCGGGCCCCCCCTCGAGGTCGTCAACTGTAAAGTGATGCGTGC | Amplify O5_20 for cloning into pZE21_MCS1 | 65 | 3.5 min |
| 14 | O5_20_R | TCAAGCTTATCGATACCGTCATACCGTCCTACGCAGATGCT | Amplify O5_20 for cloning into pZE21_MCS1 | 65 | 3.5 min |
| 15 | F01A_6_F | CCGGGCCCCCCCTCGAGGTCCCTGCACCTTTTCTGCAC | Amplify F01A_6 for cloning into pZE21_MCS1 | 61 | 3.5 min |
| 16 | F01A_6_R | TCAAGCTTATCGATACCGTCATACCGTCAGAATAAATGTATCGGTTG | Amplify F01A_6 for cloning into pZE21_MCS1 | 61 | 3.5 min |
| 17 | F01A_9_F | CCGGGCCCCCCCTCGAGGTCAGCATGCTGGACTGTATCATC | Amplify F01A_9 for cloning into pZE21_MCS1 | 61 | 3.5 min |
| 18 | F01A_9_R | TCAAGCTTATCGATACCGTCCCGAAACGGAGCTGGAAA | Amplify F01A_9 for cloning into pZE21_MCS1 | 61 | 3.5 min |
| 19 | F01A_2Δ1 | TTATAATACTTTACGATTTATGCTGTTATCATTACGCACATTC | Generate early STOP codon in F01A_2_1 by Gibson Assembly | 61 | 7 min |
| 20 | F01A_2Δ1 | GTGCGTAATGATAACAGCATAAATCGTAAAGTATTATAACGATTATAACCAGA | Generate early STOP codon in F01A_2_1 by Gibson Assembly | 61 | 7 min |
| 21 | F01A_2Δ2 | CGCCTGCGCTCCCAGTATTAGTCCTCATCCGGTATATTCCAC | Generate early STOP codon in F01A_2_2 by Gibson Assembly | 65 | 7 min |
| 22 | F01A_2Δ2 | GAATATACCGGATGAGGACTAATACTGGGAGCGCAGG | Generate early STOP codon in F01A_2_2 by Gibson Assembly | 65 | 7 min |
| 23 | F01A_2Δ3 | CAAAACTGTCATAATGTCATATCTGCC | Generate early STOP codon in F01A_2_3 by site directed mutagenesis | 63 | 7 min |
| 24 | F01A_2Δ3 | CGAGAGATACCGCAAGGG | Generate early STOP codon in F01A_2_3 by site directed mutagenesis | 63 | 7 min |
| 25 | F01A_2Δ4 | TCTCTTTCTCAGTATGCGTGC | Generate early STOP codon in F01A_2_4 by site directed mutagenesis | 63 | 7 min |
| 26 | F01A_2Δ4 | AAGCGGAGGGAGATACAT | Generate early STOP codon in F01A_2_4 by site directed mutagenesis | 63 | 7 min |
| 27 | F01A_2Δ5 | CGTTTTCCTCAGCCAATCCTGC | Generate early STOP codon in F01A_2_5 by site directed mutagenesis | 65 | 7 min |
| 28 | F01A_2Δ5 | CCTGGAGCTGACACAGCC | Generate early STOP codon in F01A_2_5 by site directed mutagenesis | 65 | 7 min |
| 29 | pCRT7_F | CAATTCCGACGTCTAAGAAACTGGATAACCGTATTACCGCCTTTG | Amplify tetR expression regulon from pCRT7 for Gibson Assembly with pZE21_MCS1 | 64 | 3.5 min |
| 30 | pCRT7_R | CTTTGAGTGAGCTGATACCGCTCATTCAGGCTGCGCAACT | Amplify tetR expression regulon from pCRT7 for Gibson Assembly with pZE21_MCS1 | 64 | 3.5 min |
| 31 | pZE21_tetR_F | AGCGGTATCAGCTCACTCAAAG | Amplify pZE21_MCS1 backbone for Gibson Assembly with pCRT7 tetR regulon | 64 | 3.5 min |
| 32 | pZE21_tetR_R | GTTTCTTAGACGTCGGAATTGC | Amplify pZE21_MCS1 backbone for Gibson Assembly with pCRT7 tetR regulon | 64 | 3.5 min |
| 33 | AcrIIA11_KpnI | CATTAAAGAGGAGAAAGGTACCATGGCAGATATGACATTAAGACAG | Amplify AcrIIA11 with KpnI and HindII sites for cloning into pZE21_tetR | 63 | 1 min |
| 34 | AcrIIA11_HindIII | GCAGCCGGATCAAGCTTTCATTAGTCCTCCTTGAGATTCCACA | Amplify AcrIIA11 with KpnI and HindII sites for cloning into pZE21_tetR | 63 | 1 min |
| 35 | AcrIIA4_KpnI | CATTAAAGAGGAGAAAGGTACCATGAATATTAATGACTTAATTAGAGAAATC | Amplify AcrIIA4 with KpnI and HindII sites for cloning into pZE21_tetR | 58 | 1 min |
| 36 | AcrIIA4_HindIII | GCAGCCGGATCAAGCTTTCATTAGTTCAACTCACTTTTTAAGGTG | Amplify AcrIIA4 with KpnI and HindII sites for cloning into pZE21_tetR | 58 | 1 min |
| 37 | SpyCas9_targetDNA  (60-mer) | ACGTTCCAACTTTCACCATAATGAAATAAGATCACTACAGGGCGTATTTTTTGAGTTATC | Double-stranded SpyCas9 target DNA olligo, ordered with 5' 6-FAM conjugate from IDT | n/a | n/a |
| 38 | SpyCas9_targetDNA  (36-mer) | TTTCACCATAATGAAATAAGATCACTACAGGGCGTA | Double-stranded SpyCas9 target DNA olligo, ordered with 5' 6-FAM conjugate from IDT | n/a | n/a |
| 39 | ORF_a_F | TAAAGAGGAGAAAGGTACCAGTGAGCCGGAGAAAGATG | Amplify *orf_a* in Figure 4—figure supplement 3 (also named F01A_2_4) from F01A_2 for KpnI/HindIII cloning into pZE21_tetR | 71 | 30s |
| 40 | ORF_a_R | CAGCCGGATCAAGCTTTCATCTATTGTTCATCGTTTGTGC | Amplify *orf_a* in Figure 4—figure supplement 3 (also named F01A_2_4) from F01A_2 for KpnI/HindIII cloning into pZE21_tetR | 71 | 30s |
| 41 | ORF_c_F | TAAAGAGGAGAAAGGTACCAATGAAATCCTGTGGAATCTC | Amplify *orf_c* in Figure 4—figure supplement 3 (also named F01A_2_2) from F01A_2 for KpnI/HindIII cloning into pZE21_tetR | 69 | 30s |
| 42 | ORF_c_R | CAGCCGGATCAAGCTTTCATTTACGCACATTCCTCCTG | Amplify *orf_c* in Figure 4—figure supplement 3 (also named F01A_2_2) from F01A_2 for KpnI/HindIII cloning into pZE21_tetR | 69 | 30s |
| 43 | ORF_d_F | ATTAAAGAGGAGAAAGGTACATGTGCGTAATGATAACAG | Amplify *orf_d* in Figure 4—figure supplement 3 (also called F01A_2_1) from contig F01A_2 for Gibson assembly into pZE21_tetR | 65 | 30s |
| 44 | ORF_d_R | CTTTGTTAGCAGCCGGATCATTAAAGAGGAGAAAGGTACC | Amplify *orf_d* in Figure 4—figure supplement 3 (also called F01A_2_1) from contig F01A_2 for Gibson assembly into pZE21_tetR | 65 | 30s |
| 45 | CACNA1D_Fwd | CACCGGCAGGAGTATTTCAGTAGTG | Golden Gate assembly of sgRNA into mammalian SpyCas9 expression vector | n/a | n/a |
| 46 | CACNA1D_Rev | AAACCACTACTGAAATACTCCTGCC | Golden Gate assembly of sgRNA into mammalian SpyCas9 expression vector | n/a | n/a |
| 47 | EMX1_Fwd | CACCGGAGTCCGAGCAGAAGAAGAA | Golden Gate assembly of sgRNA into mammalian SpyCas9 expression vector | n/a | n/a |
| 48 | EMX1_Rev | AAACTTCTTCTTCTGCTCGGACTCC | Golden Gate assembly of sgRNA into mammalian SpyCas9 expression vector | n/a | n/a |
| 49 | CACNA1D_T7E1_ Fwd | ACAGACACACACACGGTGCT | Amplification of CRISPR-Cas targeted CANA1D locus in the human genome | 64 | 2 min |
| 50 | CACNA1D_T7E1_ Rev | TGGAGTTTCTGCTCCCATTT | Amplification of CRISPR-Cas targeted CANA1D locus in the human genome | 64 | 2 min |
| 51 | EMX1_T7E1_Fwd | GCCCCTAACCCTATGTAGCC | Amplification of CRISPR-Cas targeted EMX1 locus in the human genome | 64 | 2 min |
| 52 | EMX1_T7E1_Rev | GGAGATTGGAGACACGGAGA | Amplification of CRISPR-Cas targeted EMX1 locus in the human genome | 64 | 2 min |
| 53 | AcrIIA11a.1-gBlock | tataggGagacccaagctggctagcATGGCAGATATGACGCTTCGCCAGTTCTGCGAGCGATATCGCAAGGGTGACTTCCTCGCAAAGGATCGAGAAACTCAAATCGAGGCAGGTTGGTACGATTGGTTTTGTGATGACAAAGCCTTGGCGGGCCGATTGGCAAAAATCTGGGGGATTTTGAAGGGGATAACCTCAGATTATATCTTGGATAACTACCGCGTATGGTTCAAAAACAACTGTCCAATGGTAGGACCACTGTACGACGATGTACGCTTCGAACCGCTTGATGAAGAACAGCGAGATGAGCTCTACTTCGGCGTCGCAATCGACGATAAGAGGAGGGAAAAGAAATACGTCATATTCACTGCTCGAAATGACTATGAAAACGAGTGTGGTTTCAACAACGTGAGAGAAGTACGCCAATTTATAAATGGATGGGAAGACGAATTGAAGAACGAAGAGTTCTATAAGGCTAGGGAGAAAAAACGGCAAGAAATGGAAGAAGCCAATAACAAATTCGCAGAAATAATGCAACGGGCCGATGAGATATTGTGGAACCTGAAAGAGGACtccggacctccgaagaaaaagcgaaaggtgggatccagtggatatccctatgacgtgcccgattatgcctaaCtcgagcggccgccactgtgctgga | gBlock from IDT cloned by Gibson assembly into the mammalian Acr expression vector | n/a | n/a |
| 54 | AcrIIA11b.1-gBlock | tataggGagacccaagctggctagcATGATCACGGTTCGAGAATGGATACAAAAGTTCAACGCCGGAGATTTTGATAAACCTAACAGTGGGGCCGGCTGGTATGACTGGTTCTGCGACGACAGCCAGCTTCCAAAACGCTTGAAAGCGATGGGGAATGTTATTAAAGACATTAAAAATGACTTCATACTTGACAACTATTATGTATGGTTCAAAAACAACTGTCCCTGCTGCTACCCGCTTTATGATGATTTTCGACTCGAGCCTATAACGCGCGAGGGGGACGACGAGAAGACGCGGGTAGAACGCAGGAATGCTCTCTATTTTGGAGTAGATTGCGGGCATCCCTTTGGAAGCGAGTTTAAGTACGAGATTTTCACCGCGAGAAATGGCTATGAAGTTGAGTTCAAGTGCAAAAACAAATGGGAGGTGCTGAGGCGGATAAATGAACTGGCGACAGATTTTGAGAAATTGGAAATCtccggacctccgaagaaaaagcgaaaggtgggatccagtggatatccctatgacgtgcccgattatgcctaaCtcgagcggccgccactgtgctgga | gBlock from IDT cloned by Gibson assembly into the mammalian Acr expression vector | n/a | n/a |

**Table S8**. Acr sequences used to create the phylogenetic tree in Figure 4B and those tested in Figure 4—figure supplement 3.

| **Protein Name** | **NCBI ID** | **IMG/VR ID** | **Used in** | **Sequence** |
| --- | --- | --- | --- | --- |
| AcrIIA11a.1 (AcrIIA11) | -- | -- | functional tests, phylogenetic trees | MADMTLRQFCERYRKGDFLAKDRETQIEAGWYDWFCDDKALAGRLAKIWGILKGITSDYILDNYRVWFKNNCPMVGPLYDDVRFEPLDEEQRDELYFGVAIDDKRREKKYVIFTARNDYENECGFNNVREVRQFINGWEDELKNEEFYKAREKKRQEMEEANNKFAEIMQRADEILWNLKED |
| AcrIIA11a.2 | WP_064786071.1 | -- | functional tests, phylogenetic trees | MADMTLREFCERYRKGDFLAKDRNTQIEAGWYDWFCSDKALAGRLAKIWSILKGVTSNYILDNYRVWFKNNCPMVGPLYDDVRFEPLDEEKRDELYFGVAIDDERRDNKYIIFTARNDYEDECGFNNVREVRQFINGWEEELKNEEFYKERERKKEELKKENDRCLALLRKADEVLGKHEE |
| AcrIIA11a.3 | -- | 7000000582_____SRS019787_WUGC_scaffold_26282_____SRS019787_WUGC_scaffold_26282__gene_57340 | functional tests, phylogenetic trees | MGNEMTLRQFCERYRRGDFLSKDRDVQIEAGWYDWFCSDDALAGRLAKIWNILKGIDSDYVLDNYRVWFKNNCPCEGPLYDDVRFEPIDEDKRDELYFGVAIDCVWHDSEYAVFTARNGYETEREFSNIREVRAFINGWEDALKDEEFYQKRAEKDAAMKRLSEEADRLIKMGEDILKGYQQDSEKTQ |
| AcrIIA11a.4 | -- | 3300014553_____Ga0134451_100255_____Ga0134451_10025556 | functional tests, phylogenetic trees | MTVREFCERYRNGDFLIKDRNVQIEAGWYDWFCSDTALAGRLAKIWHILKGIDSDFILDNFRVWFKNNCPIEDPLYDDVRFEPLDESKRDKMYFGIAIDDKRNAHKYTVFSARADYKNEAGFDKVKDVRAFINGWEDALKDPLFYVRKAAIHKAQEQATKEATSGLVKCLTELNAVLEEGQNDEAGK |
| AcrIIA11a.5 | -- | 3300019372_____Ga0187904_1000109_____Ga0187904_10001093 | functional tests, phylogenetic trees | MKQMTLREFQERYRNGDFLAKDFDTQVRAGWYDWFCHTSSLSNRLKKIFKIIDGITDDWMLDNFRVWFKNNCPCEGPLYDDVRFEPLDEKLRIFQYFVVAIDDKRNDSKYVIYTARMNYEKEAGFGNVREVRAWINDWHKEFDKEGVTWLN |
| AcrIIA11a.6 | -- | 3300019371_____Ga0187896_1001961_____Ga0187896_100196114 | functional tests, phylogenetic trees | MADMTIREWQDEYRRGMFEDGDFDTQVKAGWYDWFCNDSSLKNKTEKIAKIITGITNDWMLDNFRVWFKNNCPCAGPLYDDVRFEPMDESKRDELYFVVSIDDKREDDKYCVFTARNGYEKEAGFKSIKELKEWINGWEQEVSA |
| AcrIIA11a.7 | -- | 3300010270_____Ga0129306_1003310_____Ga0129306_100331014 | functional tests, phylogenetic trees | MAEMTIRQWQDEYRRGMFDDGDFDTQVKAGWYDWFCKDESLKRKTDKFAKIINGITNDWMLDNFRIWFKNNCPCARPLYDDIRFEPMDESKRDQLYFVISIDDKREDDKYCVFTARKGYEKEAGLGNVNDVIAWINAWEQEVA |
| AcrIIA11b.1 | -- | 7000000628_____SRS015782_WUGC_scaffold_33785_____SRS015782_WUGC_scaffold_33785__gene_75489 | functional tests, phylogenetic trees | MITVREWIQKFNAGDFDKPNSGAGWYDWFCDDSQLPKRLKAMGNVIKDIKNDFILDNYYVWFKNNCPCCYPLYDDFRLEPITREGDDEKTRVERRNALYFGVDCGHPFGSEFKYEIFTARNGYEVEFKCKNKWEVLRRINELATDFEKLEI |
| AcrIIA11b.2 | OHE28210.1 | -- | functional tests, phylogenetic trees | MSKELSLRTWIEKFNQGDFDSKDLETQIKAGWYDWFCKDDSLGNKTKRMGSIVKQFKDCGKLNLDNMYVWFKNNCPLAGPLYDDFRIADIESGDTLFTIQINCFREENRYTVYGKRNDFDTPLFGTESIKELVAWMNEGWADNV |
| AcrIIA11b.3 | OHE43765.1 | -- | functional tests, phylogenetic trees | MMEKQQPLKDWIQAFNSGSFESSDVKVQIKAGWYDWFCKDSSLKNKTKRMGNIIKQIKPGGKVDLDNSYVWFKNNCPLQGSLYDDFRIADLESDVTLIVVQLNSPWHDKTYTVYERLTHYEKVVFSTDSVKELVKWLNEGWDTHV |
| AcrIIA11c.1 | WP_006572312.1 | -- | functional tests, phylogenetic trees | MSVRQWQERFRAGDFSSRDRAVQCEAGWYDWFCRDEALAGHLKRISKVVLGINAPFILDNYYVWFKNNCPMAGPLYDDVRFEPLSGERGGKHFVVSLDCPHELAKWVLYTERYGYDAPEFCSGNVRNMVQYINSMAAELEQGIQPAFILEKQAVSDYVLRHEGTCGIPVYRNGEHEFSYISRKDRQLRKVTVSGGLEALPPGYEAGQAEQHGDLYVFGTEPPILETPPGQSKAAQRKGRER |
| AcrIIA11c.2 | WP_009258904.1 | -- | functional tests, phylogenetic trees | MNEMSVRTWQERFRAGDFSSRDRAVQCEAGWYDWFCRDDALAGRLKKISSVVLGITDPFILDNYYVWFKNNCPLEGPLYDDVRFEPLTGERDGKYFLVALDSHHELIKWTLYTERYGYDAPEFCCGNVREMTAYINAMAPELAQGIQPRFVLEKAAVGEYVRQHEGKAAYSIRREGDHLFAYQSSRDWKYRTVAVSDSPENVPQGFPAERAEQHGMLYVFPSKAPALDRADYVVRRAQRRKEQTR |
| AcrIIA11c.3 | WP_054338718.1 | -- | functional tests, phylogenetic trees | MSEERISVRQWQERFRAGAYESKDLKVQCAAGWYDWFCQDQALAGRLKKISRVVMGITDPYILDNYYVWFKNNCPVVGGLYDDVRFEPLSGDRDGKYFVVSLDSLHESLRWTLFTEQYDFGAPEYGCENVRDMVKYINSMAHELEEGVIPAFTVERWAVLKYMQRHDKTVNRPVHRNGEHRFSYTTFPGKQTKNIMVVSDLKDAPPDFVPDHAEQYGSFYLYCPEDTERPAPEQKRSKPARKKEAER |
| AcrIIA11c.4 | WP_016321673.1 | -- | functional tests, phylogenetic trees | MKEEISIRQWQKQFKAGFYDSPDIHTQCGAGWYDWFCQDRALAGRLKKIAKVVMGVTNPFILDHYYIWFKNNALVSGPMYDDVRFEPLSGKRDGKYFLVRLDCAGRKKWSLFSERYGFFAPEFECGNVRGMAKYIDGIGRQFAQEIQPVFLLEKRAVEHFITQQDGLCDSIVYRAGEHCYHYKSSKNPKLRTAIAASASGPPPDGFPADQAKEFRGILVWSPDGMERDMKKEADAQKKPNLKKKEGTER |
| AcrIIA11c.5 | WP_023346767.1 | -- | functional tests, phylogenetic trees | MPDELSVRQWQEQFQAGAFERSDYATQCAAGWYDWFCQDSALAGRLKKIGRVVMGITDPFILDNYYVWFKNNCPLNGPLYDDARFEPLSGERGGKYFVVSLDSPHERMKWALVTERYGFDAPEFDCRNIRDMIRYVNSIGPELRQGIIPPFIAEKDAVTAYAQRRGEPEGLHIYRDGEHCYSYTSRQDRRKRTVLAAASLEDAPPGFVSEQAHSIKGMYVYCPEDAGIPLPDLAPQDTAKSQKRKEPER |
| AcrIIA11c.6 | WP_055271317.1 | -- | functional tests, phylogenetic trees | MTEMSVRQWQERFRAGDFSSKDRAVQCEAGWYDWFCQDDALAGRLQKLSKVVMGITDPYILDHYYVWFKNNCPLSDPLYDDIRFEPLHGDRSGKYFVVIRDSPHEAHKWTLYTGRHGFEQPEFTCGNVRDMLRHINSMAPESWRGNPPPEKAMHPPQKKRKEAER |
| -- | WP_118651841.1 |  | phylogenetic trees | MADMTLRQFCERYRKGDFLAKDRETQIEAGWYDWFCDDKALAGRLAKIWGILKGITSDYILDNYRVWFKNNCPMVGPLYDDVRFEPLDEEQRDELYFGVAIDDKRREKKYVIFTARNDYENECGFNNVREVRQFINGWEDELKNEEFYKAREKKRQEMEEANNKFAEIMQRADEILGNLKED |
| -- | WP_118617142.1 |  | phylogenetic trees | MADMTLREFCERYRKGDFLAKDRNTQIEAGWYDWFCSDKALAGRLAKIWSILKGVTSNYILDNYRVWFKNNCPMVGPLYDDVRFEPLDEEKRDELYFGVAIDDERRDNKYIIFTARNDYEDECGFNNVREVRQFINGWEEELKNEEFYKERERKKEELKKENDRCLELLRKADEVLGKHEE |
| -- | WP_118315543.1 |  | phylogenetic trees | MADMTLREFCERYRKGDFLAKDRNTQIEAGWYDWFCSDKALAGRLAKIWSILKGVTSNYILDNYRVWFKNNCQMVGPLYDDVRFEPLDEEKRDELYFGVAIDDERRDNKYIIFTARNDYEDECGFNNVREVRQFINGWEEELKNEEFYKERERKKEELKKENDRYLELLRKADEVLGKHEE |
| -- | WP_101872220.1 |  | phylogenetic trees | MADMTLREFCERYRKGDFLAKDRNTQIEAGWYDWFCSDKALAGRLAKIWSILKGVTSNYILDNYRVWFKNNCPMVGPLYDDVRFEPLDEEKMDELYFGVAIDDERRDNKYIIFTARNDYEDECGFNNVREVRQFINGWEEELKNEEFYKERERKKEELKKENDRCLALLRKADEVLGKHEE |
| -- | WP_118005709.1 |  | phylogenetic trees | MADMTLREFCERYRKGDFLAKDRKTQIEAGWYDWFCDEKALAGRLAKIWSILKGITSDYILDNYRVWFKNNCPMVGPLYDDVRFEPLDEERRDELYFGVAIDDKRREKKYIIFTARNDYEDECGFNNVRELRQFINGWEDELKNEEFYKEKERKEEELKKENDKYLALLKEAEGILKKHEE |
| -- | WP_103240931.1 |  | phylogenetic trees, Figure 4—figure supplement 3 | MSCPFQAMEGGNGMERKMALREFCGRYRKGDFKGTERAVQIEAGWYDWFCEDGELEARLEKIWEILRGIRNDYVLDNYRVWFKNNCPVEEPLYDDVRFEPLDEGRRDELYFGVSIHHGKLASGYQVFTARSGYGIEAEFGSVEDVWDFINNWEGNWDDAMLHERKEAEDGRPREMLEGILQVLNEAADMLAGHLD |
| -- | WP_118642345.1 |  | phylogenetic trees | MTEMSVRQWQERFRAGDFSSKDRAVQCEAGWYDWFCQDDALAGRLQKLSKVVMGITDPYILDHYYVWFKNNCPLSGPLYDDVRFEPLHGDRNGRYFVVIRDSPHETHKWTIYTERHGFEQPEFTCANVRDMLRHINTMAPETWRDDPQPAKTPRSPQKKRKEAER |
| -- | WP_087378810.1 |  | phylogenetic trees | MTEMSVRQWQERFRAGDFSSKDRAAQCEAGWYDWFCQDDALAGRLQKLSKVVMGITDPYILDNYYVWFKNNCPLSGPLYDDVRFEPLHGDRNGRYFVVIRDSPHETHKWTIYTERHGFEQPEFTCANVRDMLRHINSMAPETWRGDPQPAKAPRSPQKKRKEAER |
| -- | WP_009260171.1 |  | phylogenetic trees | MTEMSVRQWQERFRAGDFSSKDRAVQCEAGWYDWFCQDDALAGRLQKLSKVVMGITDPYILDHYYVWFKNNCPLSGPLYDDIRFEPLHGDRSGKYFVVIRDSPHEAHKWTLYTGRHGFEQPEFTCGNVRDMLRHINSMAPESWRGNPPPEKAMHPPQKKRKEAER |
| -- | WP_024723214.1 |  | phylogenetic trees | MTEMSVRQWQERFRAGDFSSKDRAVQCEAGWYDWFCQDVALAGRLQKLSKVVMGITDPYILDHYYVWFKNNCPLSGPLYDDIRFEPLHGDRSGKYFVVIRDSPHEAHKWTLYTGRHGFEQPEFTCGNVRDMLRHINSMAPESWRGNPPPEKAMHPPQKKRKEAER |
| -- | WP_008980755.1 |  | phylogenetic trees | MTEMSVRQWQERFRAGDFSSKDRAVQCEAGWYDWFCQDDALAGRLQKLSKVVMGITDPYILDNYYVWFKNNCPLSGPLYDDVRFEPLHGDRNGRYFVVIRDSPHETHKWTLYTERRGFEQPEFTCANVRDMLRHINSMAPESWRGNPPPEKAMRPPQKKRKEAER |
| -- | WP_044943868.1 |  | phylogenetic trees | MTEMSVRQWQERFRAGDFSSKDRAVQCEAGWYDWFCQDDALAGRLQKLSKVVMGITDPYILDHYYVWFKNNCPLSGPLYDDIRFEPLHGDRSGKYFVVIRDSPHEAHKWTLYTERHGFEQPEFTCGNVRDMLRHINSMAPESWRGNPPPEKAMHPPQKKRKEAER |
| -- | WP_021633244.1 |  | phylogenetic trees | MTEMSVRQWQERFRAGDFSSKDRAVQCEAGWYDWFCQDVALAGRLQKLSKVVMGITDPYILDHYYVWFKNNCPLSGPLYDDIRFEPLHGDRSGKYFVVIRDSPHEAHKWTLYTERHGFEQPEFTCGNVRDMLRHINSMAPESWRGNPPPEKAMHPPQKKRKEAER |
| -- | WP_122789648.1 |  | phylogenetic trees | MNEMSVREWQERFRAGDFSSRDRAVQCEAGWYDWFCRDDALAGRLKKISGVVLGITDPFILDNYYVWFKNNCPLDGPLYDDVRFEPLTGERDGKYFVVSLDSPHEHMKWALVTERYGYDAPEFECGNVRDMVKYINAIAPELARGIQPRFVQEKAAVGEYVRQHEGKSSYSIRRAGDHLFAYQSPRDWKYRTVAVSDSLENVPQGFPAEQSEQHGMLYVFPSEAPALDRADMVQRAQRRKEQTR |
| -- | WP_087265981.1 |  | phylogenetic trees | MNEMSVRTWQERFRAGDFSSRDRAVQCEAGWYDWFCRDDALAGRLKKISGVVLGITDPFILDNYYVWFKNNCPVNGPLYDDVRFEPLTGERDGKYFVVSLDSPHERMKWALVTERYGYDAPEFECGNVRDMVKYINAIAPELAQGIQPRFVLEKAAVGEYVRQHEGKSSYSIRRAGDHLFAYQSPRDWKYRTVAVSDSLENVPQGFPAEQAEQHGMLYVFPSEAPALDRADMVQRAQRRKEQTR |
| -- | WP_006574413.1 |  | phylogenetic trees | MTEMSVRQWQERFRSGDFSSKDRAVQCEAGWYDWFCQDDALAGRLQKLSKVVMGITDPYILDNYYVWFKNNCPLSGPLYDDVRFEPLRGDRNGKYFVVIRDSPHETHKWTLYTERHGFEQPEFTCANVRDMLRHINSMAPETWRGDPQPAKAPRSPQNKRKEAER |
| -- | OLA43941.1 |  | phylogenetic trees | MNEMSVRTWQERFRAGDFSSRNRAVQCEAGWYDWFCRDDALAGRLKKISGVVLGITDSFILDNYYVWFKNNCPLDGPLYDDVRFEPLTGERDGKYFVVSLDSPHEHMKWALVTERYGYDAPEFECGNVRDMVKYINAIAPELARGIQPRFVQEKAAVGEYVRQHEGKSSYSIRRAGDHLFAYQSPRDWKYRTVAVSDSPENVPQGFPAELAEQHCMLYVFPSEAPALDRADVLQRAQRRKEQTR |
| -- | WP_087265909.1 |  | phylogenetic trees | MTEMSVRQWQERFRAGDFSSKDRAVQCEAGWYDWFCQDDALAGRLQKLSKVVMGITDPYILDHYYVWFKNNCPLSGPLYDDVRFEPLHGDRNGRYFVVIRDSPHETHKWTIYTERHGFEQPEFTCANVWDMLRHINTMAPETWRGDPQPAKAPHSPQKKRKEAER |
| -- | WP_021749148.1 |  | phylogenetic trees | MTEMSVRQWQERFRAGDFNSRDLSVQCEAGWFDWFCRNDALAGRLKKLSSAVLGIKAPFILDNYYVWFKNNCPMAGPLYDDVRFEPLSGEREGKYFVITLDCPHELAKWVLYTERYGYDAPEFCSGNVRDMGQYINSMAAELEQGIQPAFLLEKRAVSKYIFRHEGEHGIPVYRDREHEFSYISRKDRQLRKVMVTDSMEALPPGYGAEQAERHGKLYVFGVEPPIPEGPTVRPKTVQRGGQER |
| -- | WP_054338718.1 |  | phylogenetic trees | MSVRQWQERFRAGAYESKDLKVQCAAGWYDWFCQDQALAGRLKKISRVVMGITDPYILDNYYVWFKNNCPVVGGLYDDVRFEPLSGDRDGKYFVVSLDSLHESLRWTLFTEQYDFGAPEYGCENVRDMVKYINSMAHELEEGVIPAFTVERWAVLKYMQRHDKTVNRPVHRNGEHRFSYTTFPGKQTKNIMVVSDLKDAPPDFVPDHAEQYGSFYLYCPEDTERPAPEQKRSKPARKKEAER |
| -- | CRY95966.1 |  | phylogenetic trees | MPDELSVRQWQERFRAGAFNVQDRYTQCKAGWYDWFCQDHALAGRLKKIGRVVMGITDPFILDNYYVWFKNNCPLNGPLYDDVRFEPLSGERGGKYFVVSLDSPHEREKWALVTERYGFDAPEFDCRDIREMVKYINSIGPELQQGIVPPFIAEKDAVTAYTRLRGEPEGIHIYRDGNHRFSYAPRRGRQMRTVLTAASLEDAPPGFVSEQAHSIKGMYVYCPEDAGLPLPDLVPQDTTKSQKRKEPER |
| -- | WP_023347629.1 |  | phylogenetic trees | MPDELSVRQWQEQFRAGAFNLQDRYTQCRAGWYDWFCQDHALAGRLKKIGRVVMGITDPFILDNYYVWFKNNCPLNGPLYDDARFEPLSGNRDGKYFVISLDSPHERMKWALVTERYGFDAPEFDCRDIREMIRYVNSIGPELQKGVIPPFIAEKDAVTAYARQRGEPEGLHIYRDGDHQYSYTSRRDRRKRTVLAAASLENAPAGFVSEQAHAVKGMYLYCPEDVGIPLPEHPENIKKSREKKGVER |
| -- | WP_106720848.1 |  | phylogenetic trees | MSKEQSLRTWIESFNKGEFDSKDLQTQIKAGWYDWFCKDESLANKTKRMGNIVKQFKDGGKVNLDKIYVWFKNNCPLAGPLYDDFRIADIESGDTLFTIQINCFREEKRYTVYGKKNDFDTSLLNTDSIKELVSWFNEGWQDNV |
| -- | WP_118225168.1 |  | phylogenetic trees | MQKSAKYMRSIFKGGKSKMRSITVREWISKFNNGEFGAGDFDTQTKAGWNDWFCPDRELASRLKKMGNLIKNIENDYILDNFFLVFYNICSIDYPLFDQARFTPFRRIADKDFEFVLSFDCPYNGYKYEICTARSDYHTEFQCNTVEELFDYLKQLTEDYKKEEMNMDCQHQK |
| -- | WP_106699806.1 |  | phylogenetic trees | MSKEQSLRTWIESFNKGEFDSKDLQTQIKAGWYDWFCKDESLVNKTKRMGNIVKQFKDGGKVNLETMYVWFKNNCPLAGSLYDDFRIADIESGDTLFTIQINCFREEKRYTIYGKKNNFDTPLYNTDSIKELVNWFNEGWQDNV |
| -- | WP_012242545.1 |  | phylogenetic trees | MEKQQLLKDLIQAFNSGSFESSDVKVQIKAGWYDWFCKDSSLKNKTKRMGNIIKQIKPGGKVDLDNSYVWFKNNCPLQGSLYDDFRIADLESDVTLIVVQLNSPWHDKTYTVYERLTHYEKVVFSTDSVKELVKWLNEGWETHV |
| -- | PKK98686.1 |  | phylogenetic trees | MEKQIKLSEWIERFKSGEFDRPDTTTQINAGWFDWFCRDTSLANKTKKMGNIIKQIKAGGKVDLETSYVWFKNNCPLNGPLYDDFRIADIETNNNLIVIQIDCVWNDSKYTVYERLDGFDKPAYKTNSSRELVKWLNKGWNE |
|  | WP_118652167.1 |  | Figure 4—figure supplement 3 (Orf_a) | MSRRKMHAYGERESGGRYILDDYDFSRNHTKAQTIKRWKRNLKKKARSERRRVEHKAMSTNDEQ |
| -- | WP_118651839.1 |  | Figure 4—figure supplement 3 (Orf_c) | MKSCGISRRTKVWNIPDEDEYWERRREEFENPPRRRSQWDDEEPPPLREDYLFLDEEQEELESKYGC  PMVELDEDELLEIVEQFVKLTPESAKWEENMQGIKYKYQEECA |
| -- | WP_118651837.1 |  | Figure 4—figure supplement 3 (Orf_d). (Final 12 aa differs from NCBI sequence) | MCVMITAKIVKYYNDYNQKAFDKTFENLDELADWIFDQMQLDYTKKPGCDFLTFPTDRFGKWYEISVRPNYGGYVYWIHEIDSESGIIFSSGKYTAGKDFCAEKVGPRGGARYLSPL |
| -- | WP_064786087.1 |  | Figure 4—figure supplement 3 (Orf_e) | MDAKNQQDGAKMVEEAVGRMCRLGMMPQVITKFRKQGTVLKSETAGILYDLNDEEKKAVADWEEESGGIVYAAILSNMVFGRCLALLYVSAEEEEWELDREDLDGRVPLAYVANLDAPDCSELGSIGIAPANGGLARTE |
| -- | WP_064786085.1 |  | Figure 4—figure supplement 3 (Orf_f) | MLEYNEQTENLMEIAMMLEQLKGESEYLFEVLTDIDSITWKQKFVDWANEFTETYEPNKDVWPGNYLEVIEGFAREKILEFAGVEDKE |
| -- | WP_064786083.1 |  | Figure 4—figure supplement 3 (Orf_g) | MDLRRAGKGIVRKGKRPSVYRIGFNDGDETELTANGINELEELWRSLCPEFECEPDSVNYVERVGYEEED |
| -- | WP_064786082.1 |  | Figure 4—figure supplement 3 (Orf_h) | MGKEYEEIKAEISVRISTEDIDDIVTTALEGGICYWCRRAEVKGKYLGEFASEQISRGGVLVLHDSVDGKKRELNKEKLLSGVKQYLEDEDKPYNILVDAEDSVGCSKGVYELDCCMVDATVADMIIQYAIFDDIIYG |
| -- | WP_064786081.1 |  | Figure 4—figure supplement 3 (Orf_i) | MEEKKIVVYVLHGFWENEFTNGCAVVDVSIDLETVMKKLDEIVESKAREYVKVQEDKAEEERGFRYFEIWDENGQSAKFYIVEQYLELSQSMMEAIAESLAKGEGK |
| -- | WP_064786080.1 |  | Figure 4—figure supplement 3 (Orf_j) | MRKVYQCEHTVPPVWWFTFKDRNALGEEIVVEFRKNENTHGKHSLPALWKRRGFIDKEPETWWGVQTYVTDVQGRCSGKYNPTTKDGKLNFEWLLEATEENQQKIIDEIYRRANAIWYREDWYLEDLEEAIRSTGLEVTQERVDKLLEECHRIFDDKSERNEMLVQKASELFEEE |
| -- | WP_064786079.1 |  | Figure 4—figure supplement 3 (Orf_k) | MFGRLILETYVQDRCRDVKFKDEHLTWFEIKKNDAKRIVKRMGWESLADFLNNYTWDDTEILYQIADSCGMIVADWIEREVEDGRN |
| -- | WP_064786077.1 |  | Figure 4—figure supplement 3 (Orf_m) | MNALVIYRSLLSERDKNEFGYPEWDAAQKMLWVFIEKALEAGEESIADEIVDELYSLNDCGCTLEDKAVKADLEMLEKYGFGSRADKVRELCWE |
| -- | WP_064786075.1 |  | Figure 4—figure supplement 3 (Orf_n) | MAKRSRANRTEKATYQNIRNEHKFIDVVHHGDGHYYIIQYIKHEFPERTVVNYMGTRCGRKQKFRIGKGTLLSILEDYKKVEEA |
| -- | WP_064786073.1 |  | Figure 4—figure supplement 3 (Orf_o) | MTKQEFEKRIGAEISQKDYSIVEHVYTWHPSISEVEGKEQIAELYKSFGMPIIKNMMEAANYAETLDRAMTQAQRQVEELRKRIIRVAKGDLVVEQCITEAKKLFETVNDPHEWDVAVSYLKKRYGADAADEAIKIEHLEM |
| -- | NZ_FCFA01000076.1 nt: 57508-57359 |  | Figure 4—figure supplement 3 (Orf_p) | MADRSNSRLNEEIESKIRQWDGTVFGVSLKNMYENGTSYEGICEYADIDY |
| -- | NZ_FCFA01000076.1 nt: 56786-56613 |  | Figure 4—figure supplement 3 (Orf_r) | MQDMKVALFTIEDLKKNHPDYYRRLNPKCQVCQNILSSSKCDMCEDFDMFARAKEEMK |
| -- | WP_064786070.1 |  | Figure 4—figure supplement 3 (Orf_s) | MRQAEFAKLSREVMPVLDKLTEIAGQHGTAEKLVSITLSAEGYIHFTVHDSGMCLSRLKREDAPELEIRKQLSQEMGREEN |
| -- | WP_082923072.1 |  | Figure 4—figure supplement 3 (Orf_t) | MHRSWKSENSYPRKWEERRTDMASLNVKTEYSEYKDCKLRVGKYVEDNSVAVEIYNRWDGPIARVTTCLCDHSLAEDEAYVDTNNCPWAVALLEENGFAERTGRTRRSGYCEYPAMKFDRSKMAEFEEES |

**Works Cited**

Clemente, J.C., Pehrsson, E.C., Blaser, M.J., Sandhu, K., Gao, Z., Wang, B., Magris, M., Hidalgo, G., Contreras, M., Noya-Alarcon, O.*, et al.* (2015). The microbiome of uncontacted Amerindians. Sci Adv *1*.

Esvelt, K.M., Mali, P., Braff, J.L., Moosburner, M., Yaung, S.J., and Church, G.M. (2013). Orthogonal Cas9 proteins for RNA-guided gene regulation and editing. Nat Methods *10*, 1116-1121.

Forsberg, K.J., Patel, S., Wencewicz, T.A., and Dantas, G. (2015). The Tetracycline Destructases: A Novel Family of Tetracycline-Inactivating Enzymes. Chem Biol *22*, 888-897.

Lee, S.A., Gallagher, L.A., Thongdee, M., Staudinger, B.J., Lippman, S., Singh, P.K., and Manoil, C. (2015). General and condition-specific essential functions of Pseudomonas aeruginosa. Proc Natl Acad Sci U S A *112*, 5189-5194.

Pehrsson, E.C., Tsukayama, P., Patel, S., Mejia-Bautista, M., Sosa-Soto, G., Navarrete, K.M., Calderon, M., Cabrera, L., Hoyos-Arango, W., Bertoli, M.T.*, et al.* (2016). Interconnected microbiomes and resistomes in low-income human habitats. Nature *533*, 212-216.
